# Proton Pump Inhibitors Did Not Increase Risk of Pneumonia in Patients With Chronic Obstructive Pulmonary Disease

**DOI:** 10.14740/jocmr2322w

**Published:** 2015-09-25

**Authors:** Shou-Wu Lee, Ching-Heng Lin, Han-Chung Lien, Teng-Yu Lee, Hong-Zen Yeh, Chi-Sen Chang

**Affiliations:** a Division of Gastroenterology, Department of Internal Medicine, Taichung Veterans General Hospital, Taichung, Taiwan; b Department of Internal Medicine, Chung Shan Medical University, Taichung, Taiwan; c Department of Medical Research, Taichung Veterans General Hospital, Taichung, Taiwan; d Department of Internal Medicine, National Yang-Ming University School of Medicine, Taipei, Taiwan

**Keywords:** Chronic obstructive pulmonary disease, Pneumonia, PPI

## Abstract

**Background:**

Chronic obstructive pulmonary disease (COPD) involves the airways and pneumonia is a major cause of mortality. Proton pump inhibitors (PPIs) were found to have a positive association with pneumonia. The aim of this study was to investigate the impact of PPIs on the risk of pneumonia in patients with COPD.

**Methods:**

This was a nationwide, population-based, case-control study using data from the National Health Insurance Research Database in Taiwan. The enrolled cases were defined as patients with COPD and appearance of pneumonia between 2001 and 2005. The control group was age- and sex-matched 1:2 with the cases without pneumonia. Potential confounders such as coronary artery disease, hypertension, diabetes mellitus, heart failure, chronic kidney disease, and prescriptions of glucocorticoids over 2 weeks, were included in the analysis. Prescriptions for PPIs were identified and entered into the analysis.

**Results:**

A total of 10,131 COPD patients, including 3,377 cases with pneumonia and 6,754 without, were identified. There were 213 (5.3%) and 436 (6.5%) cases with concurrent PPIs in the two groups, respectively, and the risk of pneumonia was similar (aOR = 0.96; 95% CI: 0.83 - 1.10). Further subgroup analysis found no differences for younger patients (younger than 70 years old; aOR = 1.04; 95% CI: 0.83 - 1.10), elderly patients (older than 70 years old; aOR = 0.96; 95% CI: 0.81 - 1.15), short-term use of PPIs (less than 30 days; aOR = 1.12; CI: 0.53 - 2.34), medium-term use of PPIs (30 - 90 days; aOR = 0.86; CI: 0.72 - 1.03), or long-term use of PPIs (longer than 90 days; aOR = 0.97; CI: 0.81 - 1.15).

**Conclusion:**

PPIs did not contribute to a greater occurrence of pneumonia in COPD patients compared with non-users in this population-based case-control study. Further research is required.

## Introduction

Chronic obstructive pulmonary disease (COPD) involves the airways and is characterized by airflow limitation. Typical symptoms of COPD are dyspnea, chronic cough, and sputum production, and less common symptoms include wheezing and chest tightness [1, 2]. Pneumonia is a major cause of death in COPD patients, with mortality rates as high as 11% [3]. Therefore, preventing pneumonia is vital to reducing morbidity and mortality in COPD patients.

Proton pump inhibitors (PPIs), introduced into clinical practice in 1988, became the mainstay of therapy for many acid-related gastrointestinal disorders [4]. Previous observational studies found a positive association between PPI use and community-acquired pneumonia [5-8]. However, the relationship between PPIs and pneumonia in patients with COPD has not been determined. The aim of this study was to provide formal evidence of the impact of PPIs on the risk of pneumonia in individuals with COPD.

## Patients and Methods

This was a nationwide, population-based, case-control study. Claims data were obtained from the National Health Insurance Research Database (NHIRD) in Taiwan. The NHI provides coverage for over 98% of the island’s population of approximately 23 million. The enrolled cases in this study were defined as patients with COPD (International Classification of Diseases, ninth revision, clinical modification code 493) between 2000 and 2005, and age older than 30 years. A diagnosis of pneumonia (ICD-9-CM codes 480 to 486) in the enrolled cases during this period was recorded.

A total of 3,377 (33.3%) individuals with pneumonia were identified during the period 2000 - 2005, and they were then age- and sex-matched 1:2 with 6,754 cases without pneumonia who served as the control group.

The following potential confounders were entered into the analysis, as follows: coronary artery disease (CAD) (ICD-9-CM codes: 410 to 414), hypertension (ICD-9-CM codes 401 to 405), diabetes mellitus (DM) (ICD-9-CM codes 250), heart failure (HF) (ICD-9-CM codes 428.00), and chronic kidney disease (CKD) (ICD-9-CM codes 585, 586, 588.8, 588.9, 250.4, 274.1, 403, 404, 404 and 440.1). Concurrent prescriptions of glucocorticoids over 2 weeks that could potentially confound the association between PPI use and pneumonia were also identified. The data files contained information on patients’ prescriptions for PPIs, including omeprazole, esomeprazole, lansoprazole, pantoprazole, and rabeprazole.

Chi-square test was used to compare baseline characteristics of each categorical variable. Multivariate Cox’s regression was used to examine the influence of PPIs on pneumonia in individuals with COPD, and the odds ratios (OR) with 95% confidence interval (CI) were reported. A two-tailed P value of < 0.05 was considered statistically significant. All statistical analyses were performed using SPSS v.18.0 for Windows (SPSS, Inc., Chicago, IL, USA).

## Results

A total of 10,131 patients with COPD were identified, which included 3,377 cases with pneumonia and 6,754 without. Table 1 lists the demographic characteristics, medical conditions, and medication use of each group of patients. The distributions of age and gender between these two groups were not significantly different. There were higher prevalence rates of preexisting diseases in patients with pneumonia than in those without, including CAD (29.9% vs. 26.9%, P = 0.002), hypertension (54.1% vs. 47.9%, P = 0.001), DM (25.1% vs. 20.0%, P = 0.001), HF (11.8% vs. 8.8%, P = 0.001) and CKD (4.5% vs. 3.2%, P = 0.002), or concurrent prescriptions of glucocorticoid use (32.2% vs. 25%, P = 0.001).

**Table 1:** Number of Baseline Characteristics in Newly Identified COPD Patients in 2000 - 2005

Variables	Pneumonia		P-value^†^
	No (n = 6,754), n (%)	Yes (n = 3,377), n (%)	
Age (years)			1.000
Mean ± SD	68.3 ± 12.6	71.1 ± 13.9	
30 - 39	212 (3.1)	106 (3.1)	
40 - 49	400 (5.9)	200 (5.9)	
50 - 59	818 (12.1)	409 (12.1)	
60 - 69	1,182 (17.5)	591 (17.5)	
≥ 70	4,142 (61.3)	2,071 (61.3)	
Sex			1.000
Women	2,204 (32.6)	1,102 (32.6)	
Men	4,550 (67.4)	2,275 (67.4)	
Comorbidities			
Hypertension	3,237 (47.9)	1,827 (54.1)	< 0.001
DM	1,351 (20.0)	846 (25.1)	< 0.001
HF	591 (8.8)	398 (11.8)	< 0.001
CAD	1,816 (26.9)	1,009 (29.9)	0.002
CKD	218 (3.2)	151 (4.5)	0.002
Glucocorticoids	1,686 (25.0)	1,087 (32.2)	< 0.001
Drug use			1.000
PPI	436 (6.5)	213 (6.3)	

^†^Chi-square test. CAD: coronary artery disease; CKD: chronic kidney disease; COPD: chronic obstructive pulmonary disease; DM: diabetes mellitus; mean ± SD: mean ± standard derivation; n: numbers; PPI: proton pump inhibitor.

Among the enrolled patients with pneumonia, 213 (5.3%) cases were found to be using concurrent PPIs. Similarly, there were 436 (6.5%) cases with concurrent PPI use in the without pneumonia subgroup. The rates of concurrent PPI use between with and without pneumonia groups were not significantly different.

The strength of the association between medical history of PPIs and pneumonia is shown in Table 2. After adjustment for measured potential confounders, including concurrent prescriptions of glucocorticoids and comorbidities, the risk of pneumonia was similar whether the patients had used concurrent PPIs or not (adjusted OR = 0.96; 95% CI: 0.83 - 1.10).

**Table 2:** Odds Ratios and 95% Confidence Interval of Pneumonia Associated With Risk Factors in Conditional Multivariate Logistic Regression Analysis

Drug use	OR (95% CI)	OR^†^ (95% CI)
None	1.00 (Reference)	1.00 (Reference)
PPI	1.00 (0.87 - 1.15)	0.96 (0.83 - 1.10)

^†^Adjusted for age, sex, glucocorticoids use, and comorbidities. CI: confidence interval; OR: odds ratio; PPI: proton pump inhibitor.

With respect to age, as shown in Table 3, the prescriptions of PPIs had no effect on pneumonia in the younger patients, defined as younger than 70 years old (adjusted OR = 1.04; 95% CI: 0.83 - 1.10). Similarly, no effect of PPI prescriptions on the risk of pneumonia was seen in elderly cases, defined as 70 years old or older (adjusted OR = 0.96; 95% CI: 0.81 - 1.15).

**Table 3:** Odds Ratios and 95% Confidence Interval of Pneumonia Associated With Risk Factors in Conditional Multivariate Logistic Regression Analysis by Age

Drug use	OR (95% CI)	OR^†^ (95% CI)
Age < 70		
None	1.00 (Reference)	1.00 (Reference)
PPI	1.07 (0.85 - 1.35)	1.04 (0.83 - 1.31)
Age ≥ 70		
None	1.00 (Reference)	1.00 (Reference)
PPI	0.97 (0.81 - 1.15)	0.96 (0.81 - 1.15)

^†^Adjusted for age, sex, glucocorticoids use, and comorbidities. CI: confidence interval; OR: odds ratio; PPI: proton pump inhibitor.

Table 4 shows the effect of duration of PPIs prescriptions. There were no significant associations between PPI use and the risk of pneumonia for prescriptions less than 30 days (adjusted OR = 1.12, CI: 0.53 - 2.34), 30 - 90 days (adjusted OR = 0.86, CI: 0.72 - 1.03), or longer than 90 days (adjusted OR = 0.97, CI: 0.81 - 1.15).

**Table 4:** Odds Ratios and 95% Confidence Interval of Pneumonia Associated With Risk Factors in Conditional Multivariate Logistic Regression Analysis by Drug Dosage

Drug use	OR (95% CI)	OR^†^ (95% CI)
None	1.00 (Reference)	1.00 (Reference)
PPI ≤ 30 days	1.07 (0.51 - 2.24)	1.12 (0.53 - 2.34)
PPI 30 - 90 days	0.90 (0.76 - 1.08)	0.86 (0.72 - 1.03)
PPI > 90 days	1.04 (0.88 - 1.24)	0.97 (0.81 - 1.15)

^†^Adjusted for age, sex, glucocorticoids use, and comorbidities. CI: confidence interval; OR: odds ratio; PPI: proton pump inhibitor.

## Discussion

COPD is a chronic, slowly progressive disease characterized by airway obstruction [1]. Individuals with COPD are prone to gastroesophageal reflux disease (GERD), measured using both the GERD-specific questionnaire [9, 10] and 24-h esophageal pH monitoring [2, 11]. PPI is the major therapy for GERD based on its effective acid suppressive role.

Previous studies found that prescription of PPIs was associated with an increased risk of pneumonia. For example, three meta-analyses of five to nine observational studies found that PPI use was associated with an increased risk of pneumonia (OR from 1.34 to 1.65), especially for a shorter duration of exposure [4, 12, 13]. It has been hypothesized that PPIs induce overgrowth of bacteria in the stomach and subsequently increase the risk for micro-aspiration of bacteria [14]. Furthermore, PPIs also reduce the acidity of the upper aerodigestive tract by activating proton pumps in the larynx and lungs, thus resulting in increased bacterial colonization of these locations and therefore contributing to an increased incidence of pneumonia [15]. However, the basic demographic data of the enrolled cases in these studies were heterogeneous, and the cases with concurrent PPI use and pneumonia might have had worse general health status and a greater degree of immunocompromise.

Furthermore, some studies yielded conflicting results. A retrospective analysis of 31 trials of esomeprazole found a similar rate of pneumonia between those receiving PPI and those treated with placebo or a non-PPI comparator (OR = 0.94; 99% CI: 0.29 - 3.07) [16]. A systematic review and meta-analysis of seven randomized controlled trials found that the rate of PPI-associated respiratory tract infections was low (4% overall; OR: 1.42; 95% CI: 0.86 - 2.35) and the incidence of pneumonia was not specified [17].

In the present study, we investigated patients with COPD, a chronic disease in which pneumonia is a major cause of mortality. Our results found concurrent PPIs had no effect on risk of pneumonia after adjustment for measured potential confounders, including CAD, hypertension, DM, HF, CKD, and glucocorticoid use (adjusted OR = 0.96; 95% CI: 0.83 - 1.10). PPIs had no impact on risk of pneumonia in younger cases (adjusted OR = 1.04; 95% CI: 0.83 - 1.10) or in elderly cases (adjusted OR = 0.96; 95% CI: 0.81 - 1.15). Similarly, short-term (less than 30 days; adjusted OR = 1.12, CI: 0.53 - 2.34) and long-term (longer than 90 days; adjusted OR = 0.97, CI: 0.81 - 1.15) prescriptions of PPIs had no significant association with the risk of pneumonia.

This study had some limitations. First, respiratory illnesses, such as pneumonia and COPD may be misdiagnosed, with one disease being diagnosed as another very similar disease. Absence of corroborating clinical data such as culture specimens or radiographic imaging may result in a false positive or negative classification of pneumonia. Second, over-the-counter medication use was not included in the database used in the study, and data regarding the indication for acid suppression were not available in this study. Third, there were no available data on the patients’ lifestyle habits, including smoking status and alcohol consumption, which might be risk factors for pneumonia. Finally, even after adjustment for potential confounders, confounding by indication and disease severity may still be unmatched, as individuals prescribed PPIs are likely to have unobserved health characteristics that predispose them to pneumonia when compared to nonusers. Therefore, further research on the relationship between PPIs and pneumonia in patients with COPD is required.

### Conclusions

In this population-based case-control study, PPIs did not contribute to a great occurrence of pneumonia in COPD patients compared with non-users. Further research is required.
